# Response accuracy of GPT-4 across languages: insights from an expert-level diagnostic radiology examination in Japan

**DOI:** 10.1007/s11604-024-01673-6

**Published:** 2024-10-28

**Authors:** Ayaka Harigai, Yoshitaka Toyama, Mitsutoshi Nagano, Mirei Abe, Masahiro Kawabata, Li Li, Jin Yamamura, Kei Takase

**Affiliations:** 1 Department of Diagnostic Radiology, Tohoku University Hospital, 1-1 Seiryo-Machi, Aoba-Ku, Sendai, Miyagi Japan; 2 Department of Diagnostic Radiology, Tohoku University Graduate School of Medicine, 1-1 Seiryo-Machi, Aoba-Ku, Sendai, Miyagi Japan; 3 Graduate Medical Education Center, Tohoku University Hospital, 1-1 Seiryo-Machi, Aoba-Ku, Sendai, Miyagi Japan; 4 Division of Radiology, Tohoku Medical and Pharmaceutical University, 1-15-1 Fukumuro, Miyagino-ku, Sendai, Miyagi Japan; 5 Center for Radiology & Endoscopy, Department of Diagnostic & Interventional Radiology & Nuclear Medicine, University Medical Center Hamburg-Eppendorf, Martinstrasse 52, 20251, Hamburg, Germany

**Keywords:** GPT-4, Prompt, Radiology board examination, Linguistic variation, Translation quality

## Abstract

**Purpose:**

This study aims to investigate the effects of language selection and translation quality on Generative Pre-trained Transformer-4 (GPT-4)'s response accuracy to expert-level diagnostic radiology questions.

**Materials and methods:**

We analyzed 146 diagnostic radiology questions from the Japan Radiology Board Examination (2020–2022), with consensus answers provided by two board-certified radiologists. The questions, originally in Japanese, were translated into English by GPT-4 and DeepL and into German and Chinese by GPT-4. Responses were generated by GPT-4 five times per question set per language. Response accuracy was compared between languages using one-way ANOVA with Bonferroni correction or the Mann–Whitney U test. Scores on selected English questions translated by a professional service and GPT-4 were also compared. The impact of translation quality on GPT-4’s performance was assessed by linear regression analysis.

**Results:**

The median scores (interquartile range) for the 146 questions were 70 (68–72) (Japanese), 89 (84.5–95.5) (GPT-4 English), 64 (55.5–67) (Chinese), and 56 (46.5–67.5) (German). Significant differences were found between Japanese and English (p = 0.002) and between Japanese and German (p = 0.022). The counts of correct responses across five attempts for each question were significantly associated with the quality of translation into English (GPT-4, DeepL) and German (GPT-4). In a subset of 31 questions where English translations yielded fewer correct responses than Japanese originals, professionally translated questions yielded better scores than those translated by GPT-4 (13 versus 8 points, p = 0.0079).

**Conclusion:**

GPT-4 exhibits higher accuracy when responding to English-translated questions compared to original Japanese questions, a trend not observed with German or Chinese translations. Accuracy improves with higher-quality English translations, underscoring the importance of high-quality translations in improving GPT-4’s response accuracy to diagnostic radiology questions in non-English languages and aiding non-native English speakers in obtaining accurate answers from large language models.

**Supplementary Information:**

The online version contains supplementary material available at 10.1007/s11604-024-01673-6.

## Introduction

When engaging with speakers of several languages, each with varying levels of proficiency, the choice of language for questioning is crucial for obtaining high-quality answers. This principle also applies when interacting with large language models, as exemplified by Generative Pre-trained Transformer (GPT)-4, which supports chatbot-style conversations in over 95 languages via web browsers [1]. GPT models leverage their transformer architecture to recognize patterns across different languages and their performance is influenced by the amount of language-specific training data available [2]. GPT-4 demonstrated its highest accuracy on the MMLU benchmark—a comprehensive suite of multiple-choice problems covering 57 subjects—achieving an accuracy of 85.5% in English [3]. In comparison, GPT-4’s accuracy on translated MMLU questions was 83.7% in German, 79.9% in Japanese, and 80.1% in Mandarin [4].

In medicine, GPT-4 has exhibited impressive performance in medical licensing examinations and specialty board examinations in various languages including English [5, 6], French [7], German [8], Japanese [9, 10], and Chinese [11]. Specifically in radiology, GPT-4's performance in Japanese questions from the Japan Radiology Board Examination (JRBE) was reported to be near the estimated passing threshold, with weaker results compared to its performance in radiation oncology and nuclear medicine [10]. This suggests the need for effective methods to improve GPT-4’s performance in diagnostic radiology questions in non-English languages.

Few studies have explored the differences in GPT-4's performance across languages. For instance, in the 2023 Taiwanese Pharmacist Licensing Examination, GPT-4’s correct response rates were 54.4% for Chinese and 56.9% for English in the first stage, and 53.8% for Chinese and 67.6% for English in the second stage, with English consistently outperforming Chinese across all subjects [12]. Despite these findings, the effects of language selection and translation quality on GPT-4’s performance have not been thoroughly investigated.

We hypothesized that language selection and translation quality might influence GPT-4’s response accuracy to such specialized questions in diagnostic radiology, potentially enhancing the use of large language models as reliable medical information resources for non-native English speakers. In this study, the performance of GPT-4 on the JRBE questions in diagnostic radiology was evaluated using prompts in Japanese (i.e., the original questions) and using those translated into English, Chinese (Mandarin), and German using GPT-4 itself.We further investigated the effects of translation quality on GPT-4’s responses by comparing English prompts prepared by GPT-4, DeepL (a widely recognized AI translation tool), and a professional medical translation service. This aimed to uncover how language variations and translation quality affect the performance of advanced large language models in the field of diagnostic radiology.

## Materials and methods

### Question datasets

We retrospectively selected 146 multiple-choice questions, not including figures or tables, in diagnostic radiology from JRBE 2020, 2021, and 2022, with authorization from the Japan Radiological Society. Because the official answers were not disclosed, two board-certified radiologists, Y.T. with 12 years and M.K. with 7 years of diagnostic experience, independently reviewed and responded to the questions, consulting relevant literature and online resources. Answers agreed upon by both radiologists were deemed correct. Where opinions diverged, the radiologists reached a consensus through discussion.

### Categorization of questions

The questions from the JRBE used in this study were classified according to domains, question patterns, and levels of thinking. Initially, the questions were organized into distinct domains, including musculoskeletal, head and neck, neuro, chest, cardiovascular, breast, gastrointestinal, genitourinary, and others. In terms of question patterns, the questions were categorized into two groups: one-answer questions, which required selecting a single correct answer from five choices, and two-answer questions, which required selecting two correct answers from five choices. To assess the level of thinking required, this study used the principles of the Bloom Taxonomy framework for differentiating lower-order (questions requiring recall and basic understanding) and higher-order (questions requiring skills such as application, analysis, and evaluation) thinking skills [13, 14].

### Translation of questions into multiple languages using GPT-4

In September 2023, 146 diagnostic radiology questions from the JRBE (2020–2022) were translated into English, German, and Chinese (Mandarin) using GPT-4. We selected English owing to the abundance of data available in the language on the web [15]; German because of its linguistic similarities with English, with both belonging to the Germanic branch of the Indo-European language family; and Chinese owing to its resemblance to Japanese, particularly in the shared use of some Kanji characters [16]. Translation was achieved by inputting each Japanese question into the text input field of GPT-4’s web-based chat interface, along with a translation command specifying the target language.

### English translation of questions using DeepL

In July 2024, the 146 diagnostic radiology questions were translated into English using DeepL (DeepL SE, Cologne, Germany), a widely recognized AI translation tool offering free online translation services.

### Preparation of professional English translations

The questions were submitted to Editage (Cactus Communications Pvt. Ltd., Mumbai, India), a professional translation service, to ensure high-quality English translations.The accuracy of these translations, with particular emphasis on the correctness of radiological diagnostic terminology, was subsequently verified by A.H. and Y.T.

### Translation quality evaluations

The quality of GPT-4’s translations into English, Chinese, and German was evaluated by radiologists fluent in both Japanese and the target languages (A. H., L. L., and J. Y., respectively). The evaluation employed the XSTS protocol [17], a five-point scale designed for consistent human assessment of machine translation across language pairs [18]. This protocol focuses on meaning preservation, making it particularly suitable for evaluating response accuracy. In the XSTS protocol, Grade 4 allows for different levels of formality between language pairs, whereas Grade 5 requires the same style and level of formality. To simplify the evaluation, we combined Grades 4 and 5 into a single category, resulting in a four-point scale. Grade 1 represents the lowest quality, Grade 4 represents the highest, and Grade 3 indicates the threshold for acceptability.

### Collection of GPT-4’s responses for Japanese and GPT-4-translated questions

Between September 27 and October 29, 2023, the original Japanese questions and the English translations generated by GPT-4 were entered into the text input field of the web-based chat interface of GPT-4 to record the responses. This process was repeated for all of the 146 questions in all four languages, with each language set repeated five times. The number of correct responses in each language set was recorded as a score.

### Collection of GPT-4’s responses for DeepL-translated and professionally translated questions

For the English versions of questions translated by DeepL and a professional translation service, we used the OpenAI API to programmatically generate responses. A Python script was developed to interact with the API, ensuring consistency with the GPT-4 model version used in our 2023 analyses. This was necessary because the GPT-4 model was no longer available on the web-based chat interface during our investigation in August 2024. The script used the prompt: “Please answer the following question. Indicate the symbols for the choices you have selected at the end.” Responses were generated five times for each set of 146 questions, and the number of correct responses was recorded as a score.

### Statistical analyses

GPT-4’s performance was quantitatively evaluated for the set of 146 questions in diagnostic radiology across the four languages. Median scores with interquartile ranges (IQRs) over five attempts were calculated for each language set: the original Japanese questions and GPT-4 translations into English, Chinese, and German. The scores obtained for the Japanese questions and the GPT-4 translations were compared using one-way ANOVA. The Mann–Whitney U test was used to compare categorical scores between Japanese and GPT-4 English translations, as well as between DeepL and GPT-4 English translations. Bonferroni corrections were applied for multiple comparisons. Scatterplots were generated to depict the distribution of the proportions of correct responses across the five attempts in each language, categorized by year (2020: 42 questions, 2021: 52 questions, 2022: 52 questions), as well as cumulatively across the three years. Additionally, the scores for the original Japanese questions and the English translations were compared within categories defined by domains, question patterns, and required levels of thinking. The Mann–Whitney U test was used to compare the professionally translated English versions with those translated by GPT-4. Sankey diagrams were presented to visualize the changes in the counts of correct responses for each question in the five attempts between the original Japanese questions and translated versions. To assess the effects of translation quality on GPT-4’s performance, linear regression analysis was conducted for each language, using the counts of correct responses to each question across the five attempts as the dependent variable and the XSTS translation quality score as the independent variable. The significance threshold was set at a p-value of 0.05. The Sankey diagrams were created using the ChartExpo Excel add-in (ChartExpo, Houston, TX, USA). All statistical analyses and scatterplots were prepared using Prism 10 for macOS, version 10.3.0 (GraphPad Software, San Diego, CA, USA).

### Ethical considerations

This study did not involve human participants or utilize patient information; thus, it did not require approval by an Institutional Review Board.

## Results

### Comparison of performance across languages

Assessing GPT-4’s performance on all 146 questions of the original Japanese version revealed a median number of correct responses (i.e., scores) of 70 (IQR 68–72). For the English translations by GPT-4, the median score (IQR) was higher, at 89 (84.5–91.5). The scores for the Chinese and German versions translated by GPT-4 were lower, with medians of 64 (IQR 55.5–67) and 56 (IQR 46.5–67.5), respectively. One-way ANOVA with Bonferroni post hoc tests indicated significant differences among the language scores. Specifically, GPT-4’s performance was significantly higher in English than in Japanese (adjusted p-value = 0.002). There was no significant difference between Japanese and Chinese (adjusted p-value = 0.227), but the Japanese scores were significantly higher than those for German (adjusted p-value = 0.022) (Table 1).

**Table 1:** Number of correct responses by language and year. The p-values were calculated by comparing the number of correct responses for questions in Japanese with those for questions in English, Chinese, and German

Year (s)	Total number of questions per set	Numbers of correct responses (scores) (median [IQR])			
		Japanese	English	Chinese	German
2020–2022	146	70 [68–72]	89 [84.5–91.5]	64 [55.5–67]	56 [46.5–67.5]
Adjusted p-value		Reference	0.002	0.227	0.022

**Fig. 1:**
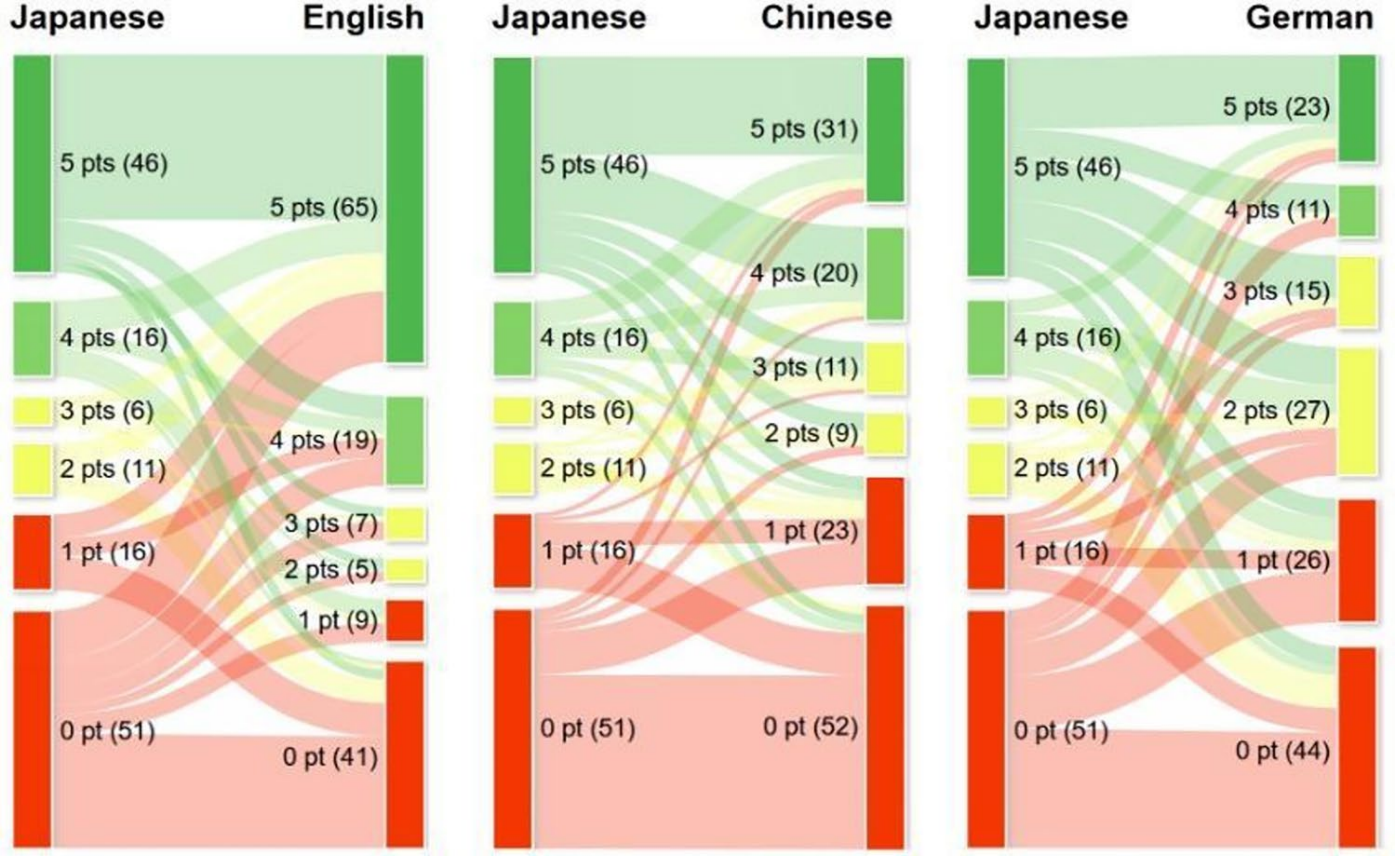
Sankey diagrams to show the differences in the count of correct responses out of five attempts (i.e., points) for each question between Japanese and the three languages used as prompts. Green nodes represent questions that earned 5 points; light green nodes, 4 points; yellow nodes, 3 or 2 points; and red nodes, 1 or 0 point

The Sankey diagrams illustrated the disparities in the counts of correct responses for each question out of five attempts (i.e., points [range: 0–5]) between the original Japanese and translated versions, showing significant variations in points (Fig. 1). These variations were particularly pronounced between the original Japanese and translated English versions. In the translation from Japanese to English, there was an increase in the number of questions with positive changes in points (≥ 4 points). However, this trend was reversed for the Chinese and German translations. The number of questions with positive changes in points ≥ 4 (from 0 to 4, 0 to 5, and 1 to 5) or negative changes in points ≤ –4 (from 4 to 0, 5 to 0, and 5 to 1) were 21 and 3 for English, 4 and 7 for Chinese, and 7 and 10 for German, respectively.

Regarding response consistency, GPT-4 selected the same answer(s) across all five attempts for 66 questions in Japanese, 84 in English, 43 in Chinese, and 36 in German out of 146 diagnostic radiology questions. On average, GPT-4 selected the same answer(s) 3.98, 4.34, 3.78, and 3.44 times in Japanese, English, Chinese, and German, respectively. In some instances, GPT-4 consistently selected the same incorrect answer(s) (Table S1), whereas in other cases, incorrect answers varied across attempts (Table S2).

### Distribution of accuracy by examination year across the four languages

**Fig. 2:**
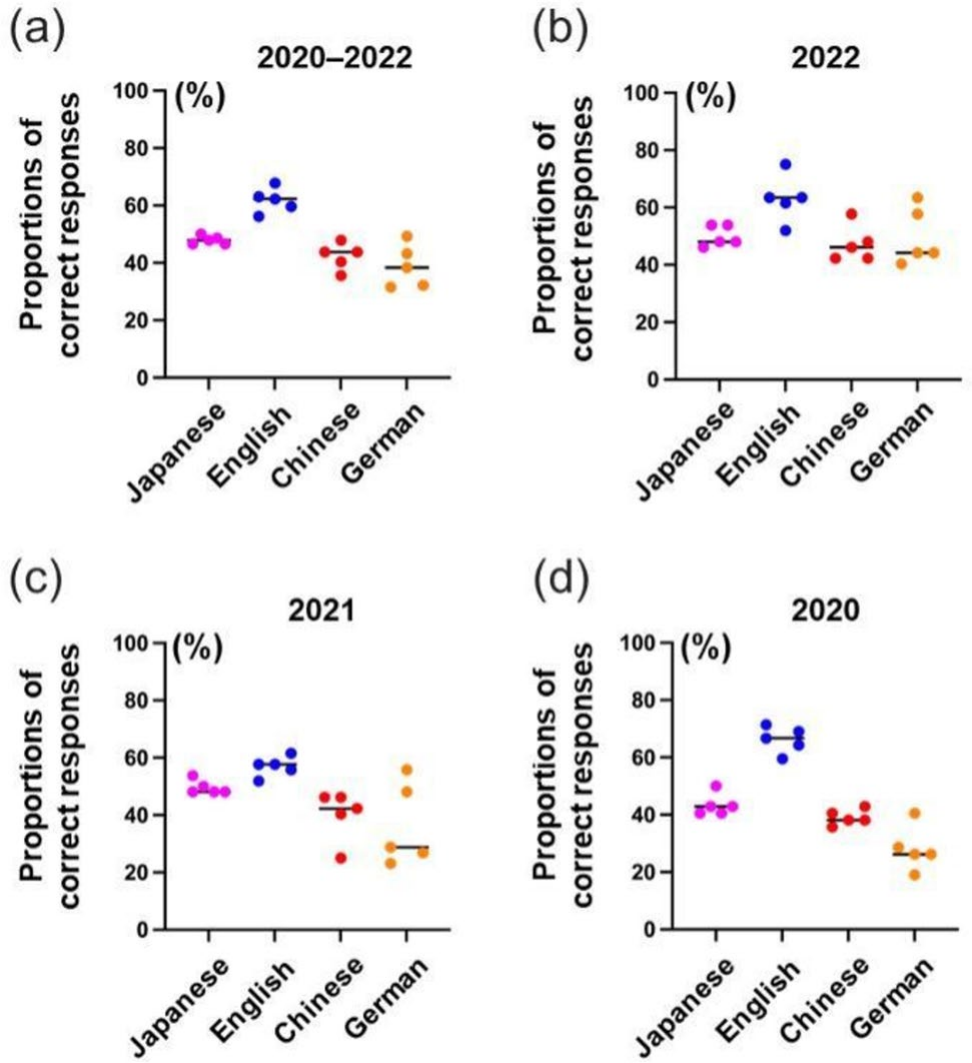
Yearly fluctuations in the proportions of correct responses (%) in five attempts by language. Panel (**a**) illustrates the proportion of correct responses, expressed as a percentage, for an aggregate of 146 Japanese Radiology Board Examination (JRBE) questions from 2020 to 2022. Panels (**b**–**d**) represent the proportions of correct responses for subsets of 52 questions from JRBE 2022, 52 questions from JRBE 2021, and 42 questions from JRBE 2020. Each plot depicts the scores for individual attempts, whereas the corresponding bars represent the median score derived from five attempts for the original Japanese and each translated version

This study categorized the 146 questions according to the year in which they were administered. To compare GPT-4’s accuracy across languages, the proportion of correct responses was calculated for each year (i.e., the score divided by the total number of questions for each respective year). Proportions, rather than raw scores, were used to construct scatterplots because the total number of questions varied across different examination years: 42 in 2020, 52 in 2021, and 52 in 2022. The scatterplots revealed variability in the proportion of correct responses per language per year. The order of accuracy, shown by the median proportions, from the highest to the lowest was English, Japanese, Chinese, and German in each year of 2020, 2021, and 2022, and over the 3-year period (Fig. 2).

### Comparison of domain-specific performance

The 146 diagnostic radiology questions tested in this study included the following domains: musculoskeletal (n = 13), head and neck (n = 9), neuro (n = 18), chest (n = 28), cardiovascular (n = 15), breast (n = 9), gastrointestinal (n = 23), genitourinary (n = 25), and others (n = 6). There were significant differences in the scores (median [IQR]) between the original Japanese (J) and English translations by GPT-4 (E) in several domains: head and neck (J 6 [6–6.5], E [7–7], p = 0.048), neuro (J 11 [10.5–12.5], E 13 [12.5–13.5], p = 0.119), chest (J 13 [12.5–15], E 17 [14.5–17.5], p = 0.048), cardiovascular (J 9 [8–9], E 13 [12.5–14], p = 0.008), gastrointestinal (J 12 [11.5–13], E 15 [13.5–15], p = 0.02), and genitourinary (J 7 [5.5–8], E 14 [13, 14], p = 0.008) domains (Table 2). In contrast to the trends observed in other domains, the musculoskeletal, neuro and breast domains did not show significant differences in scores between the original Japanese and English translations by GPT-4.

**Table 2:** Comparison of numbers of correct responses (scores) in each attempt for the original Japanese questions and the English translations by Generated Pre-trained Transformer-4 (GPT-4)

Category/Topic	Total number of questions per category	Number of correct responses (scores) Median [IQR]				Average of correct answer proportion in five attempts (%)		p-value (Scores in Japanese vs. scores in English)
		Japanese		English		Japanese	English	
Domain								
Musculoskeletal	13	5	[4.5–7.5]	4	[2.5–5.5]	44.6	30.8	0.17
Head and neck	9	6	[6–6.5]	7	[7–7]	68.9	77.8	0.048
Neuro	18	11	[10.5–12.5]	13	[12.5–13.5]	63.3	72.2	0.119
Chest	28	13	[12.5–15]	17	[14.5–17.5]	48.6	57.9	0.048
Cardiovascular	15	9	[8, 9]	13	[12.5–14]	57.3	88	0.008
Breast	9	3	[3–3.5]	5	[3.5–5]	35.6	48.9	0.087
Gastrointestinal	23	12	[11.5–13]	15	[13.5–15]	53.0	62.6	0.024
Genitourinary	25	7	[5.5–8]	14	[13, 14]	27.2	54.4	0.008
Others	6	2	[1.5–3]	3	[2.5–3]	36.7	46.7	0.40
Level of thinking								
Higher-order	99	45	[43.5–47.5]	60	[56–60.5]	45.9	59.2	0.008
Lower-order	47	24	[23.5–26]	31	[27–31.5]	52.3	63.0	0.016
Question pattern								
Two-answer	42	14	[13–18.5]	21	[18.5–23]	33.8	49.5	0.008
One-answer	104	55	[54.5–57.5]	66	[64.5–71]	53.7	64.8	0.008

The p-values were obtained by comparing the number of correct responses between Japanese and English. The proportion of correct responses in each category is also shown as a percentage for reference

### Level of thinking and question pattern comparison

Of the 146 questions, 99 were categorized as higher-order, requiring application, analysis, or evaluation, whereas the remaining 47 were categorized as lower-order, requiring recall or basic understanding. Significant differences in scores (median [IQR]) were observed between the original Japanese and English translations by GPT-4 in both categories of level of thinking: higher-order (J 45 [43.5–47.5], E 60 [56–60.5], p = 0.008) and lower-order (J 24 [23.5–26], E 31 [27–31.5], p = 0.02) (Table 2).

Questions were also categorized by pattern: two-answer (n = 42) and one-answer (n = 104). Significant differences in the scores were observed between the two language versions: two-answer (J 14 [13–18.5], E 21 [18.5–23], p = 0.008) and one-answer (J 55 [54.5–57.5], E 66 [64.5–71], p = 0.008) (Table 2).

### Effects of translation quality on GPT-4’s performance in each language

For DeepL translations, the median score was 62 (IQR: 57.5–64.5), significantly lower than the score for GPT-4 translations, which was 89 (IQR: 84.5–91.5) (p = 0.0079). The average translation grade for DeepL English translations was 2.64, significantly lower than the grade for GPT-4 translations, which was 3.12 (p < 0.0001).

Linear regression analyses showed that higher grades in translation quality were significantly associated with an increased counts of correct responses across five attempts in both the GPT-4-English, DeepL-English, and GPT-4-German versions (GPT-4: slope = 0.058, p = 0.003; DeepL: slope = 0.128, p < 0.0001; German: slope = 0.110, p < 0.0001). In contrast, for the GPT-4 Chinese translations, linear regression analyses did not reveal significant associations between translation quality and performance (slope = -0.005, p = 0.82) (Table 3).

**Table 3:** Effect of translation quality on GPT-4’s performance in each language

	GPT-4 English	DeepL English	GPT-4 Chinese	GPT-4 German
Translation grade [mean (SD)]	3.21 (0.92)	2.63 (0.95)	3.48 (0.86)	3.08 (0.88)
Counts of correct responses over five attempts (points) (n = 146) [mean (SD)]	3.02 (2.18)	2.10 (2.27)	2.12 (1.80)	2.00 (2.03)
Points in each grade category [mean (SD)]				
Grade 4	3.25 (2.07) (n = 73)	3.20 (2.24) (n = 30)	2.02 (2.04) (n = 96)	2.27 (1.80) (n = 59)
Grade 3	3.00 (2.16) (n = 39)	2.19 (2.34) (n = 52)	2.48 (2.03) (n = 33)	2.37 (2.04) (n = 43)
Grade 2	2.58 (2.44) (n = 26)	1.53 (2.07) (n = 45)	2.38 (2.39) (n = 8)	1.07 (1.17) (n = 41)
Grade 1	2.50 (2.45) (n = 8)	1.42 (2.04) (n = 19)	1.56 (1.67) (n = 9)	1.33 (1.53) (n = 3)
Simple linear regression between grades and points				
Slope	0.058	0.128	-0.005	0.110
p-value	0.003	< 0.001	0.82	< 0.001

*SD* standard deviation

### Comparison of GPT-4's Performance on English Versions Translated by GPT-4 and by a professional service

**Fig. 3:**
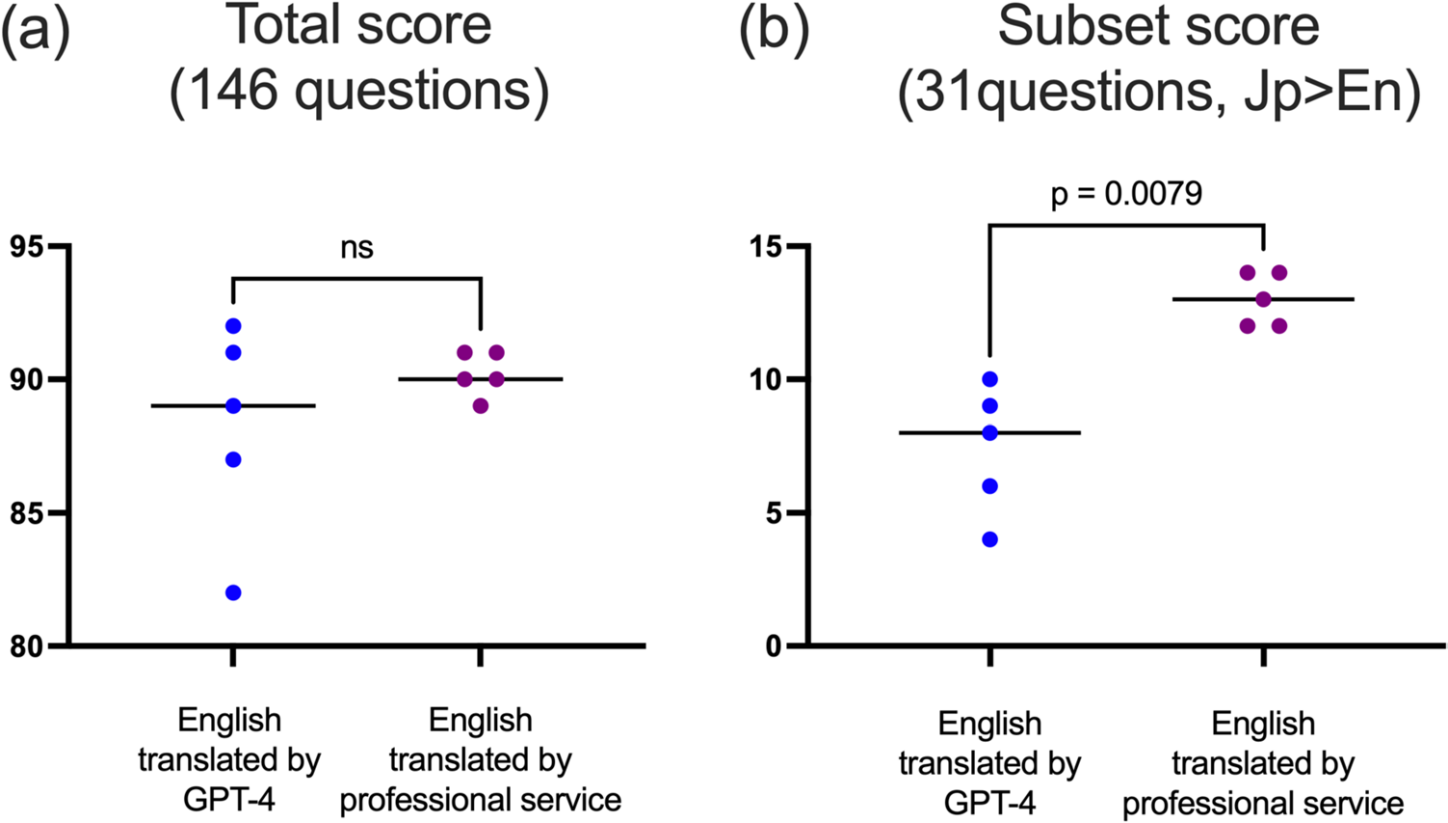
Comparison of Generative Pre-trained Transformer (GPT)-4’s scores in questions translated into English by the professional translation service compared with those translated by GPT-4. Each plot depicts the scores for each attempt, with the corresponding bars representing the median score derived from five attempts for each translation method. Panel (**a**) shows the scores for the entire set of 146 questions, whereas panel (**b**) focuses on the subset of 31 questions where GPT-4’s responses to the original Japanese questions outperformed those to the GPT-4English translations. Abbreviations: *Jp* Japanese, *En* English, *ns* not significant

**Fig. 4:**
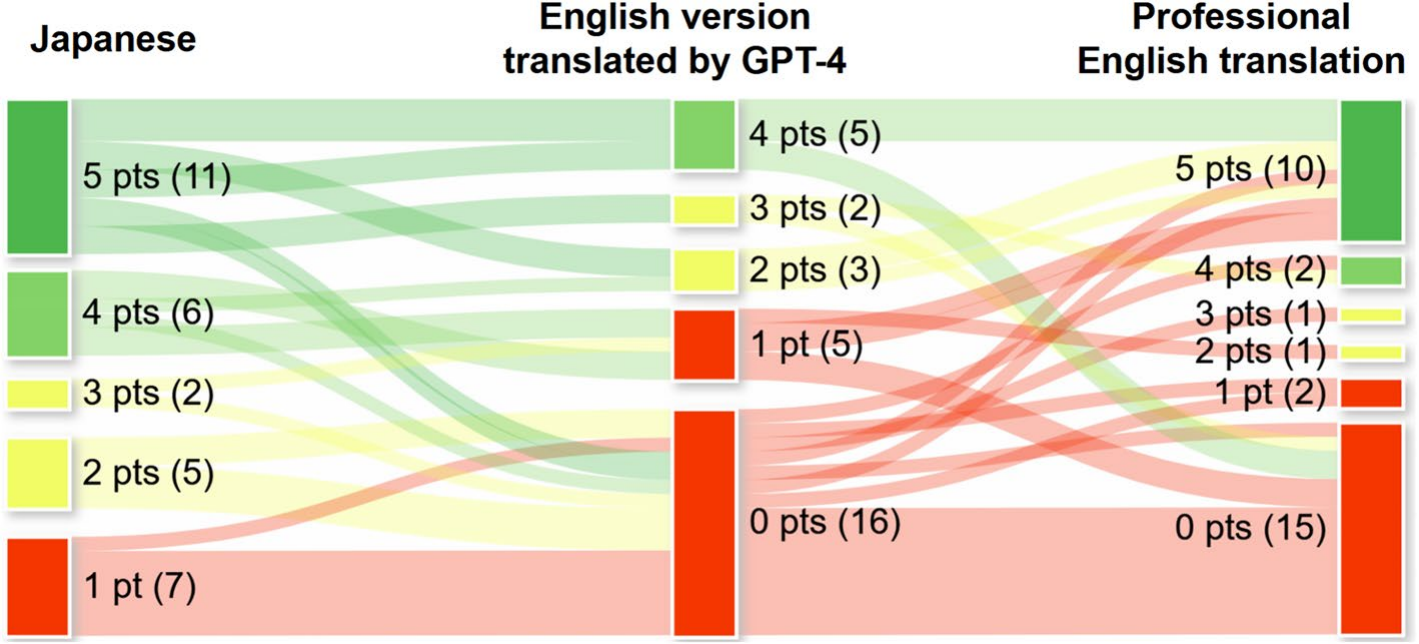
Sankey diagrams to show the differences in the count of correct responses out of five attempts (i.e., points) for each selected question among English translations by GPT-4 and professional English translation and those in Japanese. Green nodes represent questions that earned 5 points; light green nodes, 4 points; yellow nodes, 3 or 2 points; and red nodes, 1 or 0 point

The professional translations did not differ significantly from the GPT-4 translations (90, IQR: 89.5–91.0 vs. 89, IQR: 84.5–91.5) (p = 0.627) (Fig. 3a). However, a subgroup analysis of 31 questions where GPT-4’s responses to the original Japanese outperformed the English translations by GPT-4, revealed improved scores (median [IQR]) when using professionally translated questions (13 [12.0–14.0]), compared with when using GPT-4-translated questions (8 [5–9.5], p = 0.0079) (Fig. 3b). The topics of thequestions in this subset and counts of correct responses from GPT-4 across the five attempts for each question (i.e., points [range, 0–5]) are detailed in Table 4. The Sankey diagram illustrates point shifts for each question across the English version translated by GPT-4, English version translated by a professional service, and the original Japanese questions (Fig. 4). A significant increase in points from English translation by GPT-4 to professional translation was observed: a 5-point increase in 2 questions, 4-point increase in 3 questions, 3-point increase in 4 questions, 2-point increase in 0 question, and 1-point increase in 7 questions. No increase or decrease in points were observed in 10 and 5 questions, respectively.

**Table 4:** Detailed analysis results for a subset of 31 questions where GPT-4’s responses to the original Japanese outperformed the English translations generated by GPT-4

Year	Domain	Theme	Counts of correct responses over five attempts (points)			Mistranslations noted in GPT-4’s translations
			Japanese	GPT-4 English	Professional English	
2022	Musculoskeletal	Musculoskeletal disease and imaging findings	2	0	4	
2022	Musculoskeletal	Rotator cuff tears in the shoulder joint	1	0	0	“Infraspinatus” was used instead of “subscapularis”
2022	Neuro	Perfusion imaging of the brain	2	0	0	
2022	Chest	Computed tomographic findings of bronchopneumonia	2	0	1	
2022	Chest	Cytomegalovirus pneumonia	5	2	5	“Nodular shadow” was used instead of “nodular opacity”
2022	Chest	Mass-forming lung cancer	4	1	2	
2022	Breast	Mucoid carcinoma of the breast	4	1	0	“Increased echo” was used instead of “enhanced echo”
2022	Gastrointestinal	Autoimmune pancreatitis	3	1	5	
2022	Genitourinary	Prostate cancer detection in magnetic resonance imaging (MRI)	4	1	0	
2021	Neuro	Nerves passing through the superior orbital fissure	4	1	5	“Optic nerve” was used instead of “ophthalmic nerve”
2021	Neuro	Myelination in newborns and infants	2	0	0	
2021	Neuro	Imaging findings of neuromyelitis optica	5	0	5	“Optic neuritis” was used instead of “neuromyelitis optica”
2021	Neuro	MRI technique for the detection of meningitis	4	2	5	
2021	Musculoskeletal	Soft tissue calcification and ossification	2	0	0	
2021	Musculoskeletal	Charcot’s joint (neuropathic arthropathy)	5	0	0	
2021	Chest	Anatomy of the lungs	5	4	5	
2021	Chest	Lung diseases with a centrilobular distribution	5	2	5	
2021	Chest	Legionella pneumophila	5	4	5	
2021	Breast	Breast imaging with various modalities	1	0	0	
2021	Gastrointestinal	A signal reduction in a chemical shift hepatic MRI	5	4	0	
2021	Gastrointestinal	Gadolinium-ethoxybenzyl-diethylenetriamine pentaacetic acid use	5	3	4	
2021	Genitourinary	Bladder cancer and inchworm sign in MRI	1	0	0	
2020	Musculoskeletal	Imaging findings of ankylosing spondylitis	1	0	3	
2020	Musculoskeletal	Imaging findings of a bone bruise	4	0	5	
2020	Chest	Staging of lung cancer	5	3	0	
2020	Chest	Secondary pulmonary alveolar proteinosis	1	0	0	
2020	Cardiovascular	MRI findings of hypertrophic cardiomyopathy	5	4	0	
2020	Breast	Categories on mammography	1	0	0	
2020	Genitourinary	Urachal remnant	3	0	1	“Urachal fistula” was used instead of “urachal sinus”
2020	Genitourinary	Pelvic anatomy	5	4	5	
2020	Genitourinary	Urologic anatomy	1	0	0	

The table includes the examination year, domain category, question’s theme, count of GPT-4’s correct responses over five attempts (points), and any mistranslations noted in the GPT-4 translations

## Discussion

Our study assessed GPT-4’s performance in answering expert-level diagnostic radiology questions, with a particular focus on the effects of language selection and translation quality. The questions translated into English yielded significantly more accurate responses than the original Japanese ones, both overall and in most categories; however, this was not observed for the questions translated into Chinese and German. GPT-4’s performance was significantly associated with the quality of English translations, whether prepared by DeepL or by GPT-4 itself. Although refining English translations through professional editing services did not improve performance across the entire set of questions significant improvements were observed in a subset where GPT-4’s performance on GPT-4-translated questions was inferior to that on the original Japanese questions. This underscores the importance of high-quality English translations to maximize GPT-4’s response accuracy for expert-level diagnostic radiology questions in non-English languages.

The difference in GPT-4’s response accuracy across four languages can stem from the linguistic differences between Japanese and these languages and the volume of language-specific data in its training. To estimate the disparities in the available training data, the language distribution of Internet content is of interest: English, 58.8%; German, 3.7%; Japanese, 3.0%; and Chinese, 1.7% (including Mandarin and Cantonese) [15]. English and German share common roots in vocabulary and grammatical structure, whereas Japanese has a unique syntax and uses a combination of logographic (Kanji) and syllabic (Hiragana and Katakana) scripts. GPT-4’s translations from Japanese into English and German may lose nuance or include errors, affecting GPT-4’s response accuracy. GPT-4’s large volume of English training appears to compensate for translation issues, as observed in the lower performance with German translations than with Japanese translations. Employing professional English translations instead of GPT-4’s English translations improved GPT-4’s accuracy on questions, highlighting the importance of translation quality. Chinese translations exhibited numerically decreased accuracy, likely due to their minimal share of 1.7% Internet content, despite sharing some logographic characters with Japanese (Kanji). This result aligns with a previous report in which the GPT-4 response accuracy was higher in pharmacist licensing examination questions translated into English than in Chinese [12].

The inconsistencies in GPT-4's responses to the same questions likely arise from its transformer-based architecture, which generates contextual responses by predicting subsequent words and phrases based on the preceding text [19]. This model predicts each word step by step, potentially leading to variations in the final response, even when the input remains unchanged. As indicated in Table S1, GPT-4 often delivered its answers with a high degree of confidence, despite variations across different attempts. Given the discrepancies observed in our study, it is essential to use GPT-4 cautiously, especially in medical contexts where consistency and accuracy are critical.

In the domain-specific performance analysis, GPT-4 demonstrated superior accuracy with English-translated questions than with Japanese questions, except in musculoskeletal and breast radiology. In musculoskeletal radiology, the absence of a significant difference in accuracy may be owing to the intrinsic domain complexity, which involves a wide range of joints, bones, muscles, and rare malignancies [20, 21]. In breast radiology, discrepancies in mammography classification systems and terminologies, notably between the seven-tier American ACR BI-RADS ATLAS (5th ed.) [22] and the five-tier Japanese system [23], may have hindered GPT-4 response generation.

In the subanalysis of 31 questions a significant increase in points from English translation by GPT-4 to professional translation was observed as shown in the Sanky diagram (Fig. 4)This improvement can be attributed to four main factors. First, GPT-4 often confuses similar Kanji characters. For example, it translated “ophthalmic nerve (the first branch of the fifth cranial nerve)” as “optic nerve (the second cranial nerve),” likely confusing the visually similar Kanji characters for “eye” (眼) and “sight” (視). Second, GPT-4 tends to generate literal translations of medical terms, leading to incorrect terminology. For instance, it translated “posterior acoustic enhancement” observed in mucinous carcinoma as “increased echo,” which actually refers to reflection and acoustic shadowing in high echogenic structures. Third, Japanese medical terminology may not translate well into English. For example, “shadow,” an X-ray term commonly used in Japanese computed tomography (CT) terminology, is rarely used in English for CT findings. Finally, structural differences between Japanese and English, such as the omission of subjects in Japanese, often result in grammatically incomplete or ambiguous translations in English. Many of these mistranslations are easily identifiable by radiologists, highlighting opportunities to enhance GPT-4’s translation accuracy and, consequently, its response accuracy to questions in non-English languages.

This study has several limitations. First, we included JRBE questions from July 2020, 2021, and 2022, which overlap with GPT-4’s training data up to September 2022 (time of data collection), potentially influencing its performance. Second, frequent updates to ChatGPT, particularly changes in GPT models available on the web-based chat interface, made it technically challenging to replicate this study. To address this issue, we developed a Python script to consistently use the “GPT-4” model. Third, different evaluators graded the translation quality for each language, which could have introduced variability in the grades despite applying the same evaluation criteria.

In conclusion, both language selection for prompts and translation quality significantly affects GPT-4’s performance on expert-level questions for diagnostic radiology. Using English translations generated by GPT-4 can improve the accuracy of responses to these expert-level questions compared to using questions in their original Japanese form or in other languages. High-quality translation as an input can enhance GPT-4’s performance. Although GPT-4 demonstrates proficiency in processing inputs and generating outputs in multiple languages, a crucial step for non-native English speakers seeking accurate medical information in diagnostic radiology is to pose questions in high-quality English translations.

## Supplementary Information

Below is the link to the electronic supplementary material.

Supplementary file1 (DOCX 29 KB)
