# Supplementary material for: Response accuracy of GPT-4 across languages: insights from an expert-level diagnostic radiology examination in Japan
Source: Jpn J Radiol. 2024 Oct 28;43(2):319–29. doi: 10.1007/s11604-024-01673-6 (PMC11790683; doi:10.1007/s11604-024-01673-6)
Supplement: Supplementary file 1 — Supplementary file1 (DOCX 29 KB) [file 11604_2024_1673_MOESM1_ESM.docx]

**Supplementary Materials**

**Title**

**Detailed Results for Response Accuracy of GPT-4 across Languages: Insights from an Expert-level Diagnostic Radiology Examination in Japan**

**Authors:** Ayaka Harigai, Yoshitaka Toyama, Mitsutoshi Nagano, Mirei Abe, Masahiro Kawabata, Li Li, Jin Yamamura, Kei Takase

**Supplementary Table S1**

**Examples of GPT-4’s inconsistent responses with varying correct and incorrect answers across five attempts:**

| Prompt (Question) | Which of the following is not classified as T4 in the staging of lung cancer? Choose one.  a Vertebral body invasion  b Aortic invasion  c Malignant pleural effusion on the same side  d Solid part diameter >7 cm  e Additional tumor nodules in a different lobe on the same side  →**CORRECT ANSWER IS C. Malignant pleural effusion on the same side.** | | |
| --- | --- | --- | --- |
| GPT-4’s responses to the question above (Question 14 in 2020 exam) | | GPT-4’s answer | Correct or incorrect |
| Response 1 | "The TNM staging system for lung cancer is used to describe the extent and severity of the disease. The ""T"" designation refers to the size and location of the primary tumor.  Out of the given options:  a) Vertebral body invasion  b) Aortic invasion  c) Malignant pleural effusion on the same side  d) Solid part diameter >7 cm  e) Additional tumor nodules in a different lobe on the same side  The correct answer for what is NOT classified as T4 in the staging of lung cancer is:  b) Aortic invasion  Aortic invasion would typically be a sign of very advanced disease and would likely involve invasion beyond just the local structures, which would typically place it in a different category than T4." | b | Incorrect |
| Response 2 | "The TNM staging system is used to stage lung cancer, with ""T"" referring to the primary tumor, ""N"" referring to regional lymph nodes, and ""M"" referring to distant metastasis. The T stage is further divided into subcategories (e.g., T1, T2, T3, T4) based on the size and extent of the primary tumor.  From the options provided:  a. Vertebral body invasion is classified as T4.  b. Aortic invasion is classified as T4.  c. Malignant pleural effusion on the same side is classified as M1a, not T4.  d. Solid part diameter >7 cm is classified as T3.  e. Additional tumor nodules in a different lobe on the same side is classified as T4.  Therefore, the correct answer to ""Which of the following is not classified as T4 in the staging of lung cancer?"" is:  c. Malignant pleural effusion on the same side." | c | Correct |
| Response 3 | "In the TNM classification system for lung cancer, T describes the size of the primary tumor and whether it has invaded nearby structures. T4 classification includes the following:  a) Vertebral body invasion  b) Aortic invasion  c) Malignant pleural effusion on the same side  d) Solid part diameter >7 cm  e) Additional tumor nodules in a different lobe on the same side  So, based on the choices provided, none of them are incorrect for T4 classification. All the options (a through e) are features that would classify a lung tumor as T4." | None | Incorrect |
| Response 4 | "The TNM staging system is commonly used to stage lung cancer, where ""T"" stands for the size and location of the main tumor, ""N"" stands for nearby lymph nodes affected by cancer, and ""M"" stands for metastasis to distant sites.  In the context of the TNM staging for non-small cell lung cancer, T4 describes the size or location of the main tumor. Based on the choices provided:  a Vertebral body invasion - Classified as T4.  b Aortic invasion - Classified as T4.  c Malignant pleural effusion on the same side - This is actually classified as M1a, indicating distant metastasis within the thorax.  d Solid part diameter >7 cm - Classified as T4.  e Additional tumor nodules in a different lobe on the same side - Classified as T4.  Therefore, the answer is:  c Malignant pleural effusion on the same side." | c | Correct |
| Response 5 | "The TNM staging system is used to describe the anatomical extent of cancers, including lung cancer. T4 classification in lung cancer specifically addresses the size and location of the primary tumor.  From the given options:  a. Vertebral body invasion - T4  b. Aortic invasion - T4  c. Malignant pleural effusion on the same side - This is classified as M1a, not T4.  d. Solid part diameter >7 cm - T4  e. Additional tumor nodules in a different lobe on the same side - T4  So, the correct answer is:  c. Malignant pleural effusion on the same side.” | c | Correct |

**Supplementary Table S2**

**Examples of GPT-4’s inconsistent responses with varying incorrect answers across five attempts:**

| Prompt (Question) | Which of the following is less likely to show a peripheral subpleural distribution? Choose one.  a. Hypersensitivity pneumonitis  b. COVID-19 pneumonia  c. Idiopathic pulmonary fibrosis  d. Cryptogenic organizing pneumonia  e. Chronic eosinophilic pneumonia  →**CORRECT ANSWER IS A. Hypersensitivity pneumonitis presents a diffuse distribution.** | | |
| --- | --- | --- | --- |
| GPT-4’s responses to the question above (Question 16 in 2022 exam) | | GPT-4’s Answer | Correct or incorrect |
| Response 1 | "In the given options, the condition less likely to show a predominant peripheral subpleural distribution is:  e. Chronic eosinophilic pneumonia  Chronic eosinophilic pneumonia typically shows a peripheral distribution, but it's more often described as having a "photographic negative" of pulmonary edema, meaning the peripheral areas of the lungs are more involved than central areas.  For context:  a. Hypersensitivity pneumonitis - Can show a peripheral and/or centrilobular distribution.  b. COVID-19 pneumonia - Often demonstrates peripheral ground-glass opacities.  c. Idiopathic pulmonary fibrosis - Classic UIP pattern often shows peripheral, basilar predominant fibrosis.  d. Cryptogenic organizing pneumonia (formerly known as BOOP) - Can show a peripheral or peribronchial distribution.  It's always essential to rely on clinical context, patient presentation, and other imaging findings when considering these diagnoses." | e | Incorrect |
| Response 2 | "Of the options provided:  a. Hypersensitivity pneumonitis often manifests with a peripheral subpleural distribution.  b. COVID-19 pneumonia can show ground-glass opacities and consolidations with a peripheral and basal predominance.  c. Idiopathic pulmonary fibrosis (IPF) typically manifests with a peripheral subpleural distribution, with features like honeycombing, reticulations, and traction bronchiectasis in the lower lobes.  d. Cryptogenic organizing pneumonia (previously known as bronchiolitis obliterans organizing pneumonia) can also manifest with peripheral consolidations.  e. Chronic eosinophilic pneumonia typically shows peripheral airspace consolidation, especially in the upper lobes.  Given the choices, the best answer is:  b. COVID-19 pneumonia  While COVID-19 pneumonia can have a peripheral distribution, it is less classically and consistently peripheral compared to the other diseases listed. The hallmark radiographic findings for COVID-19 pneumonia are bilateral multifocal ground-glass opacities, and they can be found in various distributions, including central, peripheral, or diffuse." | b | Incorrect |
| Response 3 | "Among the choices provided, the disease less likely to show a peripheral subpleural distribution is:  b. COVID-19 pneumonia  COVID-19 pneumonia frequently shows ground-glass opacities and consolidations with a predilection for the posterior and peripheral lung regions. Still, these features aren't limited to a strict subpleural distribution, especially in advanced disease or in different phases of the disease where central and multifocal involvement can also be evident.  On the other hand:  a. Hypersensitivity pneumonitis: Often demonstrates a centrilobular and subpleural distribution with ground-glass opacities and sometimes nodules.  c. Idiopathic pulmonary fibrosis (IPF): Shows a characteristic basal and peripheral subpleural distribution with reticular abnormalities, honeycombing, and traction bronchiectasis.  d. Cryptogenic organizing pneumonia (previously known as Bronchiolitis Obliterans with Organizing Pneumonia or BOOP): Can demonstrate peripheral or peribronchovascular consolidations.  e. Chronic eosinophilic pneumonia: Typically has peripheral and upper lobe predominance with areas of consolidation.  Thus, out of these choices, COVID-19 pneumonia is less strictly associated with a peripheral subpleural distribution compared to the others. However, it's worth noting that radiologic manifestations can overlap, and clinical context is essential when interpreting imaging findings." | b | Incorrect |
| Response 4 | "The distribution of abnormalities on imaging can provide clues to specific lung diseases.  Among the choices given:  a. Hypersensitivity pneumonitis – It often shows centrilobular nodules and areas of ground-glass opacity in a peripheral and mid-to-upper lung distribution.  b. COVID-19 pneumonia – Imaging, particularly in the early stages, can show bilateral peripheral ground-glass opacities.  c. Idiopathic pulmonary fibrosis (IPF) – This condition often presents with peripheral, predominantly basal, reticular abnormalities and honeycombing.  d. Cryptogenic organizing pneumonia (COP) – It typically demonstrates patchy peripheral or peribronchovascular consolidations.  e. Chronic eosinophilic pneumonia – It is often characterized by peripheral areas of ground-glass opacity or consolidation, predominantly in the upper lobes.  Among these options, the one that is less likely to show a peripheral subpleural distribution is:  c. Idiopathic pulmonary fibrosis (IPF)  While IPF does have a peripheral distribution, it is predominantly basal and often involves the lower lobes more than the upper lobes. The other options more classically demonstrate a peripheral distribution." | c | Incorrect |
| Response 5 | "When considering the term "peripheral subpleural distribution," we refer to the tendency for lung disease to primarily affect the areas near the pleural surface, which is the membrane surrounding the lungs. Peripheral subpleural distribution refers to disease processes that predominantly involve the outer lung zones adjacent to the pleura. Different pulmonary conditions have different distribution patterns within the lung parenchyma, which can be helpful in differential diagnosis.  Out of the choices provided:  a. Hypersensitivity pneumonitis - Often shows a peripheral and peribronchovascular distribution.  b. COVID-19 pneumonia - Frequently exhibits peripheral distribution.  c. Idiopathic pulmonary fibrosis (IPF) - Predominantly demonstrates a basal and subpleural distribution.  d. Cryptogenic organizing pneumonia (COP) - Usually shows peribronchovascular and subpleural distribution.  e. Chronic eosinophilic pneumonia (CEP) - Typically displays a more central or perihilar distribution, rather than a purely peripheral one.  Therefore, option e. Chronic eosinophilic pneumonia is less likely to show a peripheral subpleural distribution compared to the other conditions listed. Always consult a healthcare professional or refer to the latest medical literature for the most accurate and current information.” | e | Incorrect |
